# Characterization of genetic and phenotypic heterogeneity of obstructive sleep apnea using electronic health records

**DOI:** 10.1186/s12920-020-00755-4

**Published:** 2020-07-25

**Authors:** Olivia J. Veatch, Christopher R. Bauer, Brendan T. Keenan, Navya S. Josyula, Diego R. Mazzotti, Kanika Bagai, Beth A. Malow, Janet D. Robishaw, Allan I. Pack, Sarah A. Pendergrass

**Affiliations:** 1 Division of Sleep Medicine/Department of Medicine, Perelman School of Medicine at the University of Pennsylvania, 125 S. 31st St, Office 2123, Philadelphia, PA 19104 USA; 2 Sleep Disorders Division/Department of Neurology, Vanderbilt University Medical Center, Nashville, TN 37232 USA; 3 Department of Psychiatry & Behavioral Sciences, University of Kansas Medical Center, Mail-Stop 4015, 3901 Rainbow Blvd., Kansas City, KS 66160 USA; 4 Geisinger Research, Rockville, MD 20852 USA; 5 Department of Biomedical Science, Charles E. Schmidt College of Medicine, Florida Atlantic University, Boca Raton, FL 33431 USA

**Keywords:** Sleep disorders, Obstructive sleep apnea, Genetics, Pleiotropy, Electronic health records

## Abstract

**Background:**

Obstructive sleep apnea (OSA) is defined by frequent episodes of reduced or complete cessation of airflow during sleep and is linked to negative health outcomes. Understanding the genetic factors influencing expression of OSA may lead to new treatment strategies. Electronic health records (EHRs) can be leveraged to both validate previously reported OSA-associated genomic variation and detect novel relationships between these variants and comorbidities.

**Methods:**

We identified candidate single nucleotide polymorphisms (SNPs) via systematic literature review of existing research. Using datasets available at Geisinger (*n* = 39,407) and Vanderbilt University Medical Center (*n* = 24,084), we evaluated associations between 40 previously implicated SNPs and OSA diagnosis, defined using clinical codes. We also evaluated associations between these SNPs and OSA severity measures obtained from sleep reports at Geisinger (*n* = 6571). Finally, we used a phenome-wide association study approach to help reveal pleiotropic genetic effects between OSA candidate SNPs and other clinical codes and laboratory values available in the EHR.

**Results:**

Most previously reported OSA candidate SNPs showed minimal to no evidence for associations with OSA diagnosis or severity in the EHR-derived datasets. Three SNPs in *LEPR*, *MMP-9*, and *GABBR1* validated for an association with OSA diagnosis in European Americans; the SNP in *GABBR1* was associated following meta-analysis of results from both clinical populations. The *GABBR1* and *LEPR* SNPs, and one additional SNP, were associated with OSA severity measures in European Americans from Geisinger. Three additional candidate OSA SNPs were not associated with OSA-related traits but instead with hyperlipidemia and autoimmune diseases of the thyroid.

**Conclusions:**

To our knowledge, this is one of the largest candidate gene studies and one of the first phenome-wide association studies of OSA genomic variation. Results validate genetic associates with OSA in the *LEPR, MMP-9* and *GABBR1* genes, but suggest that the majority of previously identified genetic associations with OSA may be false positives. Phenome-wide analyses provide evidence of mediated pleiotropy. Future well-powered genome-wide association analyses of OSA risk and severity across populations with diverse ancestral backgrounds are needed. The comprehensive nature of the analyses represents a platform for informing future work focused on understanding how genetic data can be useful to informing treatment of OSA and related comorbidities.

## Background

Obstructive sleep apnea (OSA) is defined by frequent episodes of reduced (hypopnea) or complete (apnea) cessation of airflow that occur due to upper airway obstruction during sleep, and is among the most common sleep disorders in the world [1]. When left untreated, OSA represents a significant public health burden, carrying a higher risk of serious comorbidities such as cardiovascular disease [2], cancer [3], cognitive impairment [4], and rate of progression of neurodegeneration [5]. As such, identifying more effective diagnosis and management of OSA are important research areas.

Understanding the genetic mechanisms contributing to expression of OSA and related comorbidities offers the opportunity to inform novel, personalized treatment approaches. Studies indicate that OSA is heritable [6], providing evidence for genetic influences. Genomic variation is also implicated in well-established structural risk factors for OSA (e.g., soft tissue volumes [7], craniofacial dimensions [8], and obesity [9]). Furthermore, numerous syndromes with well-defined genetic causes are associated with increased prevalence of OSA (e.g., Achondroplasia [10], Down syndrome [11], Marfan’s syndrome [12], and Prader-Willi syndrome [13]). Despite the evidence implicating genetic factors in OSA, no strong candidates are established. This is possibly due to underpowered studies, lack of replication, and the wide variability of OSA symptomatology and comorbidities within evaluated patient populations.

With the expansion of biorepositories linked to electronic health records (EHRs), large sample sizes and a variety of OSA-related information—as well as other clinical data—are available, offering unprecedented opportunity to establish more robust genetic associations. In addition, phenome-wide association studies (PheWAS) can help reveal pleiotropic genetic effects influencing expression of OSA and/or co-occurring conditions by testing variants for associations with a broad range of EHR-derived phenotypes [14]. In addition to uncovering pleiotropy, identifying candidate variants associated with OSA-related comorbidities, but not OSA itself, may suggest the original associations were due to unrecognized confounding.

Therefore, in the present study we used EHRs linked to genomic data to determine if previously reported associations between single nucleotide polymorphisms (SNPs) and OSA validated in clinical samples in the United States. We then used PheWAS to provide evidence of pleiotropic effects for OSA-associated SNPs, or to determine whether previous evidence of associations with OSA may actually reflect relationships with underlying comorbidities. This work represents one of the largest candidate studies, and the first PheWAS, focused on OSA genetics.

## Methods

### Search strategy and selection criteria for OSA-associated genomic variants

To identify OSA candidate genetic variants, PubMed was queried for original research papers and previous reviews (up to January 1, 2018) that focused on genetic risk factors for OSA. Search terms for PubMed queries were as follows: (obstructive sleep apnoea [Title/Abstract] OR obstructive sleep apnea [Title/Abstract])) AND (polymorphism OR genetic variant OR genetic association OR gene OR genome wide association study OR genome-wide association study). PubMed results were filtered to include studies conducted in humans. To further expand the query space, we additionally searched the embase resource (https://www.embase.com/) focusing on articles that were available via embase or MEDLINE. Search terms for embase queries required use of ‘sleep disordered breathing’ for disease focus, as opposed to allowing queries for ‘obstructive sleep apnea’, and were as follows: ‘sleep disordered breathing’/mj AND (‘genetic polymorphism’/mj OR ‘single nucleotide polymorphism’/mj OR ‘genetic association study’/mj OR ‘genetic association’/mj) AND ([embase]/lim OR [medline]/lim) AND [< 1966–2018]/py AND [humans]/lim AND [abstracts]/lim AND [english]/lim AND ([article]/lim OR [letter]/lim OR [review]/lim) AND ([young adult]/lim OR [adult]/lim OR [middle aged]/lim OR [aged]/lim). Abstracts were manually reviewed to ensure that results related to genetic studies that were specifically relevant to OSA, as opposed to phenotypes that may reflect something other than an OSA diagnosis (e.g., treatment response). Studies were excluded if there were no full-texts available or they represented analyses of genomic regions for which individual variants could not be identified. The remaining full manuscripts were manually reviewed to identify candidate variants. Candidate variants were then mapped to reference SNP IDs (rsIDs) and the final list of candidate SNPs was pruned for linkage disequilibrium (based on r^2^ < 0.50) separately in each independent dataset to identify SNPs tagging independent genomic regions. When necessary, proxy SNPs for candidate variants were identified using data from 1000 Genomes, phase III [15] Europeans, since this ancestral group represented the largest proportion of our analysis datasets using rAggr (http://raggr.usc.edu/) and an r^2^ > 0.50.

### Study populations

Geisinger samples were selected from the current MyCode biobank participants. MyCode is a major resource for research that combines information obtained from DNA and serum with health information from the electronic health record (Epic) and other sources intended to improve the prevention, diagnosis, and treatment of disease [16]. No specific clinics or practices are targeted by MyCode and there is a high consent rate, suggesting samples are representative of the overall health system [16]. EHR-defined cases (for definition, see below) were required to have at least 1 year of activity post their first OSA diagnosis and be between 18 and 88 years of age at time of the OSA diagnosis. EHR-defined non-cases (for definition, see below) were required to have at least 2 years of activity in the health system between January 1, 2008 and December 31, 2016 and be between 18 and 88 years of age as of December 31, 2016.

Vanderbilt University Medical Center (VUMC) samples were selected using data from individuals in Vanderbilt’s biorepository linked to electronic health records (BioVU). BioVU is a biorepository of DNA extracted from discarded blood collected during routine clinical testing and linked to de-identified medical records in the Synthetic Derivative. As BioVU samples are obtained from every clinic that collects blood for routine laboratory tests at VUMC, we expect minimal bias with regard to the clinical aspects of these samples [17]. The Synthetic Derivative is a de-identified copy of the main hospital electronic health record databases created for research purposes. De-identification was achieved primarily through the application of a commercial electronic program, which was applied and assessed for acceptable effectiveness in scrubbing identifiers. Individuals included in the study were required to have available genome-wide genotyping data and at least 2 years of activity in the health system between January 1, 2001 and June 6, 2017. EHR-defined cases were additionally required to have be between 18 and 88 years of age at time of the first code usage. EHR-defined non-cases were required to be between 18 and 88 years of age at the time of data analysis.

Analyses were performed across three independent datasets from the two sites (i.e., Geisinger European Americans [Geisinger] and Vanderbilt University Medical Center European Americans [VUMC-EA] and African Americans [VUMC-AA]). The Geisinger dataset was comprised of 39,407 individuals with European ancestry, and the VUMC datasets included 20,688 individuals with European ancestry and 3396 individuals with African ancestry (Table 1). Other ancestral groups were not included due to limited sample sizes at Geisinger and VUMC.

**Table 1:** Comparison of Demographics across Datasets and between Cases and Controls

Analysis Sample	Demographics	Totals*	EHR-defined OSA	EHR-defined No OSA	*p*-value
Geisinger (EA)	N	39,407	5760	33,647	–
	Age^a^, years	59.3 (17.3)	61.1 (12.6)	58.9 (17.2)	2.65 × 10^− 29^
	BMI^a^, kg/m^2^	31.6 (18.7)	40.1 (9.8)	30.1 (6.8)	< 1.58 × 10^− 322^
	Male, %	40.9%	52.9%	38.9%	2.15 × 10^−87^
VUMC (EA)	N	20,688	2831	17,857	–
	Age^a^, years	56.6 (17.5)	55.8 (13.0)	56.8 (18.2)	2.88 × 10^−4^
	BMI^a^, kg/m^2^	28.8 (7.3)	34.3 (9.0)	27.9 (6.6)	1.08 × 10^− 241^
	Male, %	42.4%	56.9%	40.1%	1.93 × 10^− 62^
VUMC (AA)	N	3396	331	3065	–
	Age^a^, years	47.5 (17.3)	49.2 (13.8)	47.3 (17.6)	5.73 × 10^−2^
	BMI^a^, kg/m^2^	31.0 (8.6)	38.5 (10.4)	30.2 (8.0)	2.91 × 10^− 36^
	Male, %	37.8%	41.7%	37.4%	1.37 × 10^− 1^

^a^Age and body mass index (BMI) are presented as means (standard deviations), and for cases with EHR-defined OSA were reported in the same year as the first use of the OSA-related code, while for controls the most recent measurements were used. *Traits compared between analysis datasets were all significantly different at *p* ≤ 3.37 × 10^− 7^. *EHR* Electronic health record, *VUMC* Vanderbilt University Medical Center, *AA* African or *EA* European (North) American

### Genotype quality control and imputation

Genotype data for the Geisinger dataset were generated using the Illumina® HumanOmniExpressExome bead chip. Genotype data for the VUMC datasets were generated using the Illumina® Multi-Ethnic Global Array (MEGA). Quality control (QC) procedures required DNA samples to have > 90% genotyping call rate and be unrelated based on PI-HAT≤0.05. Directly genotyped markers were required to have > 99% call rate with minor allele frequency > 0.01, and *p*-values testing significant deviation from Hardy-Weinberg Equilibrium > 1.0 × 10^− 7^. Genetically-informed ancestry was determined using principal components analysis with reference human genomes available via the 1000 Genomes Project, phase III [15]. The quality controlled data for the European dataset from Geisinger were imputed using Impute2 and reference human haplotypes available via the Haplotype Reference Consortium [18]. The quality controlled data for the European and African VUMC datasets were imputed using reference human haplotypes available via the 1000 Genomes Project, phase III [15]. Imputed genotypes were required to have info scores ≥0.3. Notably, while all other QC thresholds were required for inclusion in association tests, two imputed SNPs were missing in > 10% of the VUMC-AA dataset: rs999944 (missing = 19.3%) and rs7804372 (missing = 10.5%). These SNPs were not tested for associations in this dataset.

### EHR-Derived OSA phenotypes

Our primary OSA phenotype was cases and non-cases status derived using an EHR algorithm based on the total instances (e.g., on different dates) of OSA-related International Classification of Diseases (ICD) 9th or 10th revision codes (Table S1) in the individual’s health record [19]. This algorithm was recently validated with clinical chart reviews across multiple sites in the U.S., including Geisinger and VUMC [19]. For the present study, the minimum number of code instances that achieved positive predictive value (PPV) and negative predictive value (NPV) of at least 90% was used for determining OSA cases and non-cases. At Geisinger, a case-definition of OSA-related codes on at least 3 different dates in the EHR was required to achieve PPV and NPV ≥90% (PPV [95% CI] =94.9 [88.5, 98.3]; NPV [95% CI] = 95.0 [88.7, 98.4]) [19]. At VUMC, a case-definition of OSA-related codes on at least 2 different dates achieved these thresholds (PPV = 97.5 [92.9, 99.5]; NPV = 94.0 [87.4, 97.8]) [19]. Non-cases were defined as zero OSA-related diagnostic codes in the EHR at both sites. Individuals not meeting the case or non-case definitions were excluded.

In addition to case and non-case status, secondary analyses were performed using 13 available phenotypes from sleep study (polysomnography [PSG]) reports available for a subset of the dataset from Geisinger (*n* = 4957 EHR-defined cases and *n* = 1614 non-cases); these data were not available at VUMC due to issues with de-identification of polysomnography results that impeded inclusion of these data in the Synthetic Derivative. Only data from full-night, diagnostic in-laboratory sleep studies (e.g., not split-night studies) with at least 120 min of total sleep time were included. If an individual had more than 1 sleep report, the diagnostic sleep study that was conducted on the date closest to the first usage of an OSA code (among EHR-defined cases) or the most recent study (among EHR-defined non-cases) was chosen. Standard American Academy of Sleep Medicine criteria was used to define hypopneas. Phenotypes included respiratory indices (Apnea/Hypopnea Index [AHI], Central Apnea Index [CAI], Obstructive Apnea Index [OAI]), mean event durations (Respiratory Event [RDI] Duration and Duration of Apnea/Hypopnea), hypoxia measures (Percent Time with SaO_2_ < 89% during Non-Rapid Eye Movement [NREM] or Rapid Eye Movement [REM], Minimum SpO_2_ during Respiratory Event or Total Sleep Time), measures of sleep quality (Number of Awakenings, Sleep Efficiency, Wake After Sleep Onset), and subjective sleepiness (the Epworth Sleepiness Scale [ESS] [20]).

### EHR-derived body mass index

Body mass index (BMI) was calculated from the EHR data at each site based on available measures of height and weight. The primary BMI value of interest was the BMI closest to the time of the first OSA code usage (in EHR-defined cases) or the most recent value (for EHR-defined controls). Site-specific quality control and data cleaning procedures were applied to assure high-quality BMI measurements.

At Geisinger, height was recorded in inches and weight in pounds; there was no evidence of outliers explained by incorrect units. Calculated BMI values > 3 standard deviations above or below a patient’s median BMI value were excluded as outliers. If fewer than three BMI measurements remained after these exclusions, all BMI data were considered unreliable and excluded. At VUMC, height and weight values were previously cleaned and obvious outliers removed based on a validated algorithm [21]. Median BMI values within the same year as the first OSA code usage (for EHR-defined cases) or most recent year of EHR data (for EHR-defined non-cases) were calculated, manually reviewed, and excluded as outliers if > 3 standard deviations above or below a patient’s overall median BMI.

### Statistical analyses

Unless otherwise specified, continuous data are summarized as means and standard deviations and categorical data as frequencies and percentages. Age and BMI were compared between EHR-derived cases and non-cases in each dataset using T-tests and across the Geisinger, VUMC-EA and VUMC-AA datasets using analysis of variance and Tukey-Kramer *post-hoc* pairwise comparisons. Sex was compared using chi-squared tests. All models were evaluated adjusting for age and BMI at the time of first OSA code usage (for EHR-defined cases) or most recently measured (for EHR-defined non-cases), as well as sex (except in sex-stratified analyses). Given the higher genetic diversity of the VUMC sample, all analyses were additionally adjusted for global ancestry using the first three principal components calculated for each dataset, as was done previously for VUMC datasets [22]. Principal component adjustment was not included in analyses of the Geisinger dataset, given the high genetic homogeneity of the sample and the fact that previous studies have not observed a difference in results when adjusting for ancestry PCs compared to unadjusted for PCs in this dataset [16].

### Genetic associations with OSA-related phenotypes

Analyses were performed to evaluate the associations between candidate SNPs identified from the systematic literature review and EHR-derived OSA phenotypes. SNPs were coded additively with respect to the minor allele for the population being assessed. To maintain consistency throughout, all results are reported based on mapping to the positive strand. Primary analyses evaluated associations between candidate SNPs and EHR-based OSA diagnosis using logistic regression models, separately within each dataset. In addition to independent analyses within each sample, we performed two separate meta-analyses of the two European American datasets alone and of all three analysis samples (Geisinger, VUMC-EA, and VUMC-AA). Inverse-variance, standard error based meta-analyses, and the possibility of heterogeneity among the studies, was tested in the METAL software package (version: 2011-03-25) [23]. Given a priori evidence of associations between candidate SNPs and OSA, significant evidence of validation was set at an uncorrected *p* < 5.0 × 10^− 2^ when the initial study reporting the association with OSA was conducted in an ancestral population of comparable ancestry to our study populations. Significant evidence of generalizability was determined using the Benjamini-Hochberg (BH) method, and was based on a false discovery rate of 5% (e.g., q < 5.0 × 10^− 2^) when the initial study reporting the association with OSA was conducted in an ancestral population of different ancestry than our study populations. To determine if age, sex or BMI modified the associations between any candidate SNP and EHR-derived OSA diagnosis, we performed interaction tests by including a product term (SNP x [covariate]) in a logistic regression model that also included both main effects (SNP, covariate). To control for the numerous interactions tested in these analyses, statistical significance was determined using the BH method and was set at q < 5.0 × 10^− 2^.

Secondary analyses of genetic associations with 13 OSA phenotypes from sleep study reports in the Geisinger European American sample were performed using linear regression. Given the quantitative nature of these traits and the comparatively smaller analysis sample, sleep traits were analyzed in all patients with available data regardless of EHR-defined case/control status. As the majority of these quantitative traits did not meet normality assumptions, Box-Cox power transformations were applied and then Z-scores were calculated prior to analyses. For variables that included values of 0, half of the minimum non-zero value was added to each observation prior to transformation. Given a priori evidence of associations between candidate SNPs and OSA, we determined significance for results separately for SNPs where the initial study reporting the association was conducted in an ancestral population of comparable ancestry to our study populations versus a different ancestry. Specifically, when the initial study was conducted in either Europeans or African Americans, *p*-values from association tests between each SNP and all traits were corrected using BH and significant evidence of validation was set at q < 5.0 × 10^− 2^. When the initial study was conducted in non-comparable ancestral populations, significant evidence of generalizability was determined based on correction of *p*-values from association tests between all of these SNPs and all phenotypes.

### Phenome-wide association study methods for OSA candidate SNPs: discovery (Geisinger) and replication (VUMC)

To determine whether there was evidence of pleiotropic genetic effects of OSA candidate variants with other phenotypes in the EHR or whether initial associations between SNPs and OSA may be driven by underlying associations with co-occurring conditions (e.g., unmeasured confounding), we performed a phenome-wide association study (PheWAS).

Given that the PheWAS were novel analyses, as opposed to the validation studies that were conducted for previously reported OSA candidate SNPs, we chose to perform a discovery analysis in the Geisinger dataset and replication in the VUMC-EA dataset. We also tested for generalization to the VUMC-AA dataset. For discovery PheWAS analyses in the Geisinger sample, associations between all OSA candidate SNPs and a total of 574 non-OSA ICD codes and 143 Logical Observation Identifier Names and Codes (LOINC) mapped median laboratory values were assessed using logistic and linear regression models, respectively. For non-OSA ICD codes, only codes resulting in ≥200 cases in the Geisinger dataset were included (Table S2). For each ICD code, ‘cases’ were defined as individuals with codes used on ≥3 different dates and ‘controls’ were defined as individuals with absence of this code or a code within the same hierarchy. Individuals with one or two instances of a code were excluded from the analysis for that code. Box-Cox power transformations were applied to quantitative median laboratory values prior to analysis. Only laboratory values available for ≥1000 individuals [24] in the Geisinger dataset were included (Table S3). Statistical significance in these discovery analyses was determined using the BH method and was set at q < 5.0 × 10^− 2^.

Significant SNP-phenotype associations discovered in the Geisinger sample were replicated in the VUMC datasets using codes that reflected the same phenotype code (PheCode Map 1.2 [25]) as the discovery ICD code, present on ≥3 different dates in the individual’s record. PheCodes were used for replication analyses to circumvent issues related to potential differences in specific ICD code usage across the two clinical sites, as they represent distinct health care systems [25]. Prior to PheCode mapping, all ICD-10 codes were mapped to ICD-9 codes using CMS General Equivalency Mapping via the Agency for Healthcare Research and Quality MapIT Tool, Application Version 5.1.110, data version 2.2018.110X (https://www.qualityindicators.ahrq.gov/resources/Toolkits.aspx). Given the goal of replicating specific SNP-phenotype associations discovered in the Geisinger sample, the significance threshold for these analyses was set at *p* < 5.0 × 10^− 2^. All PheWAS tests were conducted while adjusting for age, sex and BMI as described above. In addition, analyses conducted in the VUMC datasets were conducted while also adjusting for ancestry PCs.

## Results

### Literature-derived identification of OSA candidate variants

The initial queries of PubMed identified 428 studies meeting the search criteria, and 221 of these met inclusion criteria after manual reviews of abstracts. Forty articles were also identified by focusing only on studies sourced from embase and MEDLINE; however, cross-referencing search results indicated that only one of these was not also included in PubMed and captured in the initial queries. Ultimately, a systematic review of 205 studies revealed 51 unique OSA candidate SNPs (Fig. S1 and Table S4). Five SNPs were dropped due to low allele frequencies (MAF < 0.01) and six SNPs were dropped due to missingness. Analyses described below evaluated associations with 40 SNPs: 38 directly measured in our samples, and two proxy SNPs for literature-derived SNPs rs35424364 (proxy: rs7752028, r^2^ = 0.97) and rs25531 (proxy: rs11080123, r^2^ = 0.50).

### Sample characteristics

Demographic characteristics of the analysis datasets, overall and stratified by EHR-derived OSA status, are presented in Table 1. Among the Geisinger sample, the mean (SD) age was 59.3 (17.3) years and the mean (SD) BMI was 31.6 (18.7) kg/m^2^. A total of 9.5% of the Geisinger sample met Center for Disease Control criteria for severe obesity (BMI ≥ 40 kg/m^2^), 40.9% were male and 14.6% met EHR-defined criteria for OSA. Individuals with EHR-defined OSA were older, had higher BMIs and were more likely to be male than those with no evidence of OSA (Table 1). Among the VUMC-EA sample, the mean (SD) age was 56.6 (17.5) years and the mean (SD) BMI was 28.8 (7.3) kg/m^2^; 7.5% of the dataset met criteria for severe obesity, and 42.4% were male. In total, 13.7% of individuals met EHR criteria for OSA. These individuals had higher BMI and were more likely to be males, but were 1 year younger on average than those with no evidence of OSA. Among the VUMC-AA sample, the mean (SD) age was 47.5 (17.3) years and the mean (SD) BMI was 31.0 (8.6) kg/m^2^, 13.8% met criteria for severe obesity, 37.8% were male and 9.7% met EHR-defined criteria for OSA. Individuals with EHR-defined OSA had significantly higher BMI than non-cases, but there were no significant differences in age or gender.

As shown in Table 1, participants in the Geisinger dataset had higher BMIs than both VUMC datasets. The VUMC-EA dataset had the lowest average BMI and the highest proportion of males, while the VUMC-AA dataset was the youngest and had the lowest proportion of males.

### Associations of OSA candidate variants with EHR-derived OSA diagnosis

**Fig. 1:**
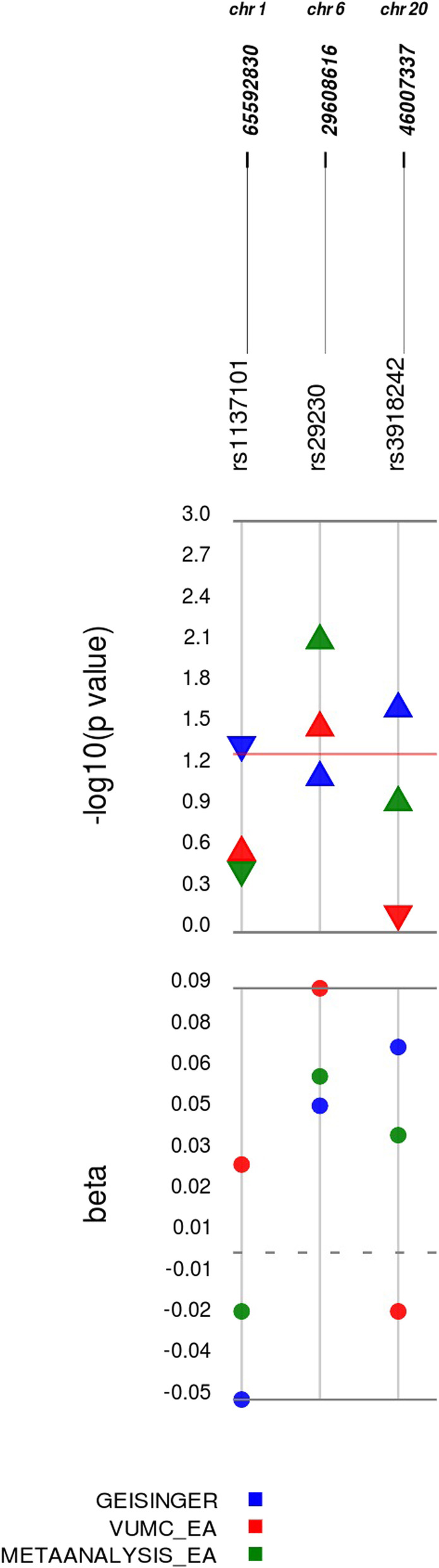
Validated Associations with Obstructive Sleep Apnea Diagnosis in European Americans. Plotted are the estimates of the additive effects (beta) of the minor allele at each candidate SNP associated with a validated definition of OSA obtained from the EHR, along with the corresponding -log_10_*p*-value. For each SNP, the rsID, chromosome and hg38 base pair location are provided. Results from analyses conducted in the Geisinger European American (EA) dataset are plotted in blue, VUMC European Americans in red, and meta-analyses of results from tests conducted in both European American datasets in green. For *p*-values, up arrows denote increased risk for OSA diagnosis given the minor allele at this SNP and down arrows denote reduced risk. Red line denotes unadjusted significance threshold (*p* < 5.0 × 10^− 2^)

Associations between candidate SNPs and EHR-derived OSA case and non-case status were evaluated in each dataset. Of the 40 SNPs tested, SNPs in the *LEPR* gene (rs1137101; *p* = 3.78 × 10^− 2^) and ~ 2 kb upstream of the *MMP-9* gene (rs3918242; *p* = 2.65 × 10^− 2^) validated in the Geisinger sample and a SNP in the *GABBR1* gene (rs29230; *p* = 3.65 × 10^− 2^) validated in the VUMC-EA sample (Fig. 1 and Table 2). The variant in *GABBR1*, but not variants in *LEPR* and *MMP-9*, was also significantly associated with EHR-derived OSA status in the meta-analysis of both European American datasets (*p* = 8.44 × 10^− 3^). No previously reported candidate SNPs validated in the EHR-derived African American dataset from VUMC (Table S4).

**Table 2:** Validated Associations with Obstructive Sleep Apnea Diagnosis and Severity in European Americans

SNP	Chr	Closest Gene	EHR-Derived Trait	Effect Allele	Geisinger		VUMC		Meta-Analysis	
					β (SE)	*p*-value†	β (SE)	*p*-value†	β (SE)	*p*-value†
rs1137101	1p31.3	*LEPR*	OSA Diagnosis	G	− 0.05 (0.02)	3.78 × 10^−2^	0.03 (0.03)	2.96 × 10^− 1^	− 0.02 (0.02)‡	3.09 × 10^− 1^
			WASO (mins)		−0.07 (0.02)	2.64 × 10^−4^				
			Sleep Efficiency (%)		0.06 (0.02)	2.28 × 10^−3^				
			Awakenings (#)		−0.05 (0.02)	9.40 × 10^− 3^				
rs29230	6p22.1	*GABBR1*	OSA Diagnosis	G	0.05 (0.03)	8.42 × 10^−2^	0.09 (0.04)	3.65 × 10^−2^	0.06 (0.02)	8.44 × 10^−3^
			WASO (mins)		0.08 (0.02)	1.72 × 10^−3^				
rs3918242	20q13.12	*MMP-9*	OSA Diagnosis	T	0.07 (0.03)	2.65 × 10^−2^	−0.02 (0.04)	6.48 × 10^−1^	0.04 (0.03)	1.28 × 10^−1^

Shown are results for literature-derived SNPs where OSA associations reported in populations of European ancestry validated for an association with electronic health record-derived OSA-related traits in European Americans. †*p*-values are unadjusted. ‡Indicates evidence for significant heterogeneity (*p* < 5.0 × 10^−2^). Abbreviations: *SNP* Single nucleotide polymorphism, *Chr* Chromosome, *EHR* Electronic health record, *SE* Standard error, *OSA* Obstructive Sleep Apnea, *Resp* Respiratory, *WASO* Wake After Sleep Onset, *Mins* Minutes. All SNPs evaluated in EHR-based datasets are mapped to the positive strand. More details and FDR-adjusted q-values for OSA diagnosis association tests are available in Table S4 and sleep study report variables tests in Table S6

In addition to assessing overall associations between candidate SNPs and EHR-derived OSA diagnosis, we evaluated whether there was evidence that associations were modified by age, sex or BMI using statistical interaction tests. These analyses suggested that associations between 15 SNPs and OSA diagnosis may be modified by age, sex or BMI (Table S5). However, none of the strata-specific associations were significant after correction for multiple comparisons.

### Associations of OSA candidate variants with quantitative sleep traits

**Fig. 2:**
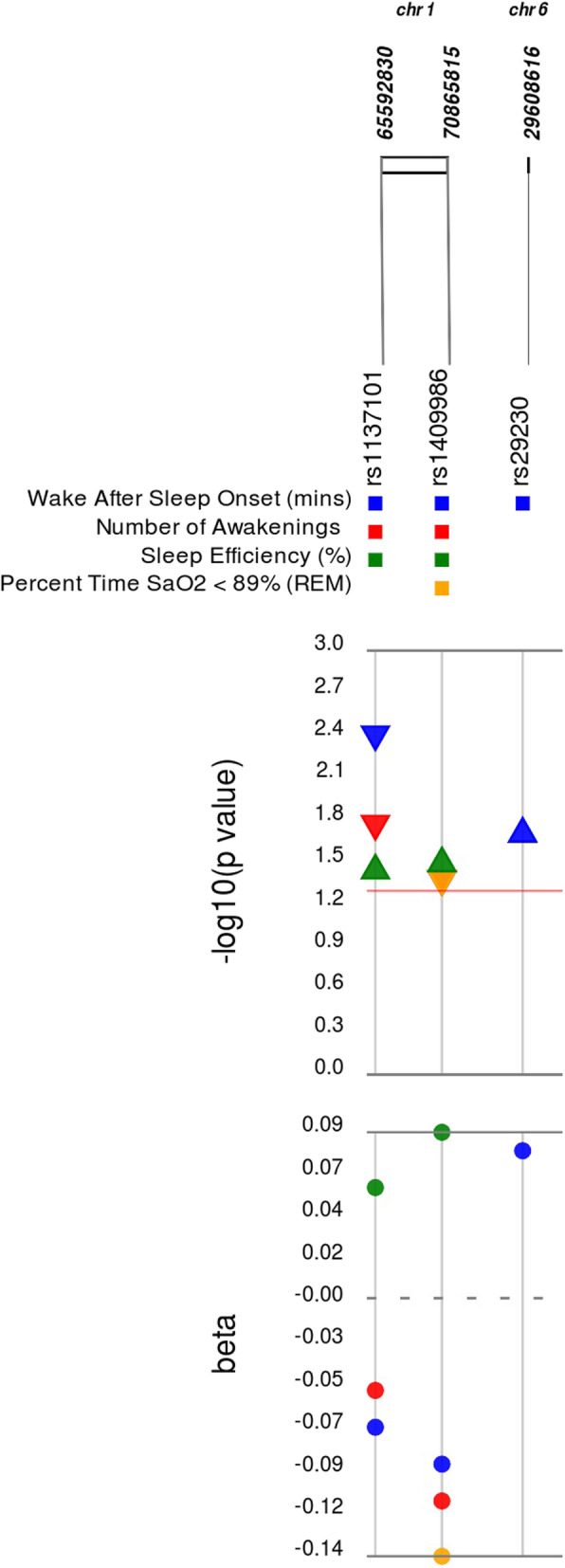
Associations between Literature-derived Candidate SNPs and Sleep Study Report Variables. Plotted are the estimates of the additive effects (beta) of the minor allele at each candidate SNP reported to be associated with OSA in populations of European ancestry on variables obtained from sleep study reports available in the Geisinger dataset only, along with the corresponding –log_10_*p*-value. For each SNP, the rsID, chromosome and hg38 base pair location are provided. Colors of betas and *p*-values reflect those obtained for the corresponding sleep study report variable listed above. For *p*-values, up arrows denote increases in variable measurements and down arrows denote decreases. Red line denotes unadjusted significance threshold (*p* < 5.0 × 10^− 2^)

In addition to associations with EHR-derived OSA diagnosis across samples, we examined relationships between candidate SNPs and quantitative OSA phenotypes available in a subset of the European American sample at Geisinger (Table S6). Three SNPs were significantly associated, following multiple testing corrections, with quantitative measures of OSA severity obtained from sleep study reports at Geisinger (Fig. 2). Of particular interest, the *GABBR1* and *LEPR* SNPs associated with EHR-derived OSA diagnosis (Table 2) were both associated with quantitative OSA phenotypes. The *GABBR1* SNP (rs29230) associated with increased risk for EHR-defined OSA diagnosis in meta-analysis was associated with increased wake after sleep onset. Additionally, the *LEPR* gene variant (rs1137101) associated with reduced risk for OSA diagnosis was associated with a decreased number of awakenings and wake after sleep onset, and an increased sleep efficiency (Table 2). One additional SNP, which was not associated with EHR-defined OSA diagnosis, was associated with sleep study report variables. This SNP, a non-coding variant in *PTGER3* (rs1409986) previously related to increased risk for OSA, was associated with fewer awakenings, less percent time with SaO_2_ < 89% in REM and decreased wake after sleep onset.

### Phenome-wide association studies of OSA candidate variants

In addition to analyses of the relationship between candidate OSA SNPs and OSA-related phenotypes, to assess for possible pleiotropy or whether previous relationships with OSA may be driven by underlying associations with OSA-related comorbidities, we performed a PheWAS for each candidate SNP. None of the three SNPs that validated for an association with OSA diagnosis were associated with other non-OSA clinical traits once we controlled for multiple testing (Table S7). However, seven other candidate SNPs were associated with non-OSA clinical traits at an FDR < 5% in discovery analyses at Geisinger (Table S7). Of these, associations between three SNPs and non-OSA clinical traits replicated in the VUMC-EA dataset (Table 3). In particular, rs7412, the marker of the *APOE-ε2* allele, was associated with lower risk of hyperlipidemia in both the Geisinger and VUMC-EA samples. Moreover, rs429358, the marker of the *APOE-ε4* allele, was associated with increased risk of hyperlipidemia in all of the EHR-derived datasets. These associations validate previously reported relationships between *APOE* SNPs and hyperlipidemia [26]. Finally, a variant in the *HLA-DQA1* gene (rs2187668) was associated with increased risk for celiac disease and autoimmune diseases of the thyroid in both European American datasets. The association between rs2187668 and EHR-derived codes for celiac disease validates results from previous studies [27].

**Table 3:** Associations between Obstructive Sleep Apnea Candidate SNPs and Other EHR-derived Clinical Traits

SNP	Chr	Closest Gene	EHR-Derived Trait	Effect Allele	Geisinger (EA)		VUMC (EA)	VUMC (AA)		
					β (SE)	q-value	β (SE)	q-value	β (SE)	q-value
rs2187668	6p21.32	*HLA*	Celiac Disease	T^a^	1.62 (0.12)	5.80 × 10^−38^	1.15 (0.34)	2.83 × 10^−3^	Non-varying phenotype/genotype^c^	
			Hypothyroidism NOS		0.15 (0.03)	7.67 × 10^−4^	0.22 (0.08)	1.13 × 10^−2^	0.44 (0.24)	3.10 × 10^−1^
rs429358	19q13.32	*APOE*	Hyperlipidemia	C	0.26 (0.03)	7.57 × 10^−20^	0.16 (0.04)	3.86 × 10^−4^	0.36 (0.11)	4.69 × 10^−3^
			HDL-Cholesterol^b^		−1.51 (0.15)	8.80 × 10^− 19^	−5.95 (1.35)	1.82 × 10^−4^	−2.21 (2.62)	5.59 × 10^− 1^
			Mixed hyperlipidemia		0.17 (0.04)	3.97 × 10^−3^	0.20 (0.05)	1.93 × 10^−4^	0.39 (0.11)	4.69 × 10^−3^
rs7412	19q13.32		Hyperlipidemia	T	−0.46 (0.03)	9.88 × 10^−46^	−0.21 (0.06)	1.01 × 10^−3^	− 0.22 (0.14)	3.42 × 10^−1^

Shown are phenome-wide association test results where presence of the clinical code for the respective disorder on ≥3 different dates in the EHR was associated with the OSA candidate SNP at FDR-corrected q < 5.0 × 10^−2^ in the Geisinger dataset, and replicated in the Vanderbilt University Medical Center European American (EA) dataset. ^a^Literature-reported effect allele located on the negative strand. ^b^Indicates an association with the median laboratory value for this measure in serum or plasma. ^c^Indicates test could not be conducted due to non-varying phenotype or genotype in either cases or controls. Abbreviations: *SNP* Single nucleotide polymorphism, *Chr* Chromosome, *EHR* Electronic health record, *SE* Standard error, *AA* African American, *NOS* Not otherwise specified, *HDL* High-density lipoprotein. All SNPs evaluated in EHR-based datasets are mapped to the positive strand. See Table S7 for more details

## Discussion

By harnessing the rich resources available in electronic health records linked to genetic data, we performed a comprehensive study of the relationship between genetic variants previously implicated in OSA risk and both EHR-defined OSA status and other phenotypes derived from data in the medical record. A majority of the candidate SNPs evaluated did not show significant associations with EHR-derived OSA status in our samples, suggesting these original results could be false positive associations. However, three of the candidate variants validated among the European American samples, including variants within or near the *LEPR*, *MMP-9* and *GABBR1* genes. In particular, variants in *GABBR1* and *LEPR* were associated with both OSA diagnosis and severity in the EHR-derived European American datasets evaluated and may be particularly likely to translate to clinical populations of European ancestry. Ultimately, results reported here represent a first step towards leveraging EHR-based phenotypes to tease apart the heterogeneity underlying expression of OSA. We expect this may help provide the basis for more personalized treatment, tailored to particular symptom and comorbidity profiles in OSA.

### EHR-derived evidence supports influences of SNPs in GABBR1 and LEPR on OSA diagnosis and variables from sleep studies

Arguably, the *GABBR1* variant (rs29230) had the most consistent evidence for an association with OSA-related traits in European Americans in our sample, including a significant association with increased risk for OSA in meta-analysis (OR = 1.06, *p* = 8.44 × 10^− 3^) and evidence of an association with increased wake after sleep onset (*p* = 1.73 × 10^− 3^). The association between this SNP and OSA was reported in a candidate gene study conducted in 174 individuals from Turkey, where OSA was defined as apnea hypopnea index (AHI) ≥5 and controls were confirmed using medical histories [28]; the SNP was associated primarily among males [28]. A subsequent study of 266 individuals from China confirmed the association of this SNP with OSA [29]. *GABBR1* encodes a receptor for the main inhibitory neurotransmitter in humans, gamma-aminobutyric acid (GABA). *GABBR1* variation may influence risk for OSA by affecting activity of the encoded receptor, which is expressed in hypoglossal motor neurons innervating the tongue, resulting in altered inhibition of tongue movement [28, 29]. The predicted consequences of this SNP vary across different in silico algorithms, with some reporting the variant to be damaging and others benign (Ensembl Variant Effect Predictor, https://uswest.ensembl.org); thus, additional functional characterization of this SNP is necessary to elucidate a mechanistic role in OSA risk.

There was also a missense variant in *LEPR* (rs1137101) that had substantial evidence for a relationship with OSA in European Americans at Geisinger, including decreased risk for EHR-defined OSA (OR = 0.95, *p* = 3.78 × 10^− 2^) and associations with increased sleep efficiency (*p* = 2.28 × 10^− 3^), reduced wake after sleep onset (*p* = 2.64 × 10^− 4^), fewer awakenings (*p* = 9.40 × 10^− 3^) and shorter respiratory event duration (*p* = 3.01 × 10^− 2^). Despite this evidence, the variant was not associated in the VUMC-EA dataset or in joint meta-analysis. Previous meta-analysis of seven candidate gene studies observed an association between rs1137101 and decreased risk for OSA specifically in Europeans, but not in Asians [30]. *LEPR* encodes a receptor for the adipocyte-specific leptin hormone and there is evidence of a relationship between leptin levels and OSA [30]. Higher levels of leptin have been observed in OSA patients compared to controls [31], and the AHI has been shown to be an independent predictor of the evening/morning leptin ratio, suggesting that OSA might affect leptin diurnal rhythms [32]. Given the relationship between OSA and obesity, it is notable that this SNP is not associated with risk for obesity [33–35], suggesting *LEPR* genetic variation has effects on OSA that are independent of this established pathway.

Among variants associated with quantitative EHR phenotypes, one SNP demonstrated inconsistent evidence when compared to previous literature. The A allele at the rs1409986 SNP, located in a non-coding transcript of the *PTGER3* gene, was previously associated with increased OSA risk—defined based on AHI ≥ 15—in an extensive candidate gene study of 2904 individuals of European ancestry [36]. In our analyses, the rs1409986 SNP was not associated with OSA diagnosis. Additionally, the previous study failed to replicate the association between rs1409986 and OSA risk in analyses of independent datasets, indicating that this association may not generalize across distinct analysis datasets [36]. Furthermore, differences in definitions of PSG-based OSA diagnosis could explain why an association with EHR-defined OSA diagnosis did not validate. The study reporting the original association between rs1409986 and OSA defined case status as an AHI ≥ 15 [36], while to qualify for a diagnosis of OSA based on EHR-derived clinical codes it was necessary to have clinical codes used on more than one date in the EHR. Clinical codes for OSA in the EHR are likely used once in order to obtain a diagnostic sleep study, and a second or third code would then be used following a PSG with an AHI meeting the American Academy of Sleep Medicine criteria for OSA (i.e., AHI > 5).

While not associated with OSA diagnosis, we did observe associations between the rs1409986 SNP and less time spent at low oxygen saturation, decreased number of awakenings and increased sleep efficiency. The original study reporting this association evaluated log-transformed AHI, but not other measures from the PSG, and did not observe a significant relationship that survived multiple testing correction (uncorrected p  =  2.0 × 10^− 2^) between genotypes at the rs1409986 SNP and log-transformed AHI [36]. Notably, PSG-derived measures of sleep quality (e.g., number of awakenings, sleep efficiency, wake after sleep onset) are not specific to OSA and are more often used to define insomnia. As such, it is difficult to know whether our results reflect evidence that this SNP is associated with less severe symptoms of OSA, or insomnia. It is also notable that both the *GABBR1* and the *LEPR* variant were associated with measures of sleep quality in addition to OSA-related traits. Interestingly, our group has observed a distinct subgroup of individuals with OSA who have insomnia-related symptoms [37]. These associations may reflect genetically-distinct phenotypic subgroups of OSA with comorbid insomnia.

### Associations of OSA candidate SNPs may reflect underlying comorbidities

Beyond OSA diagnosis and severity, we evaluated associations between OSA candidate SNPs and other EHR-based phenotypes, which can provide evidence of pleiotropic genetic effects or indicate that original associations were driven by genetic effects on OSA-related comorbidities. Several SNPs previously associated with OSA showed significant associations with EHR phenotypes. Of particular interest were SNPs tagging the *APOE-*Ɛ2 (rs7412) and *APOE-*Ɛ4 (rs429358) alleles, as well as a variant in the *HLA* gene (rs2187668). Specifically, the *APOE-*Ɛ2 tagging SNP previously shown to be protective for OSA was not associated with EHR-defined OSA diagnosis. Notably, rs7412 was instead associated with a lower risk of hyperlipidemia in both Geisinger and VUMC-EA. It is feasible that prior associations with decreased risk of OSA may be driven by this underlying association, as patients with OSA are at higher risk for metabolic syndrome [38]. Similarly, while the *APOE-*Ɛ4 tagging SNP (rs429358) previously shown to increase OSA risk was not associated with EHR-defined OSA in our sample, this SNP was associated with increased risk for hyperlipidemia and lower levels of HDL cholesterol. These associations are supported by prior literature [26], which observed that *APOE-*Ɛ2 carriers had smaller LDL with normal HDL, and *APOE-*Ɛ4 carriers had smaller HDL. Once again, it is feasible that the original association was driven by the effect of this *APOE* variant on underlying cardio-metabolic disease, rather than a true association with OSA itself.

Finally, the *HLA* SNP previously implicated in OSA risk was not associated with OSA-related phenotypes in our sample, but showed effects on increased risk for autoimmune thyroid diseases and celiac disease. Hypothyroidism is considered a risk factor for OSA, with as many as 35% of individuals having comorbid or eventual OSA [39]. Potential mechanisms include deposition of mucoprotein in the upper airway, reduced neural output to airway musculature, abnormal ventilatory control, and a dual relationship with obesity [40]. Similarly, a connection between OSA and celiac disease has been proposed [41]. Specifically, symptoms of celiac disease (e.g. gastroesophageal reflux disease) may contribute to disturbed sleep; however, evidence suggests that sleep disorders in individuals with celiac disease are independent of gastrointestinal issues [41]. Furthermore, celiac disease and OSA share common features of lymphatic hyperplasia and local inflammation, and studies conducted in children report an increased prevalence of OSA in patients with celiac disease [42]. There was no mention in the original study reporting an association between this SNP and OSA of excluding individuals with thyroid disorders or celiac disease [43]. As such, it is possible that a proportion of OSA cases in the original study had underlying comorbidities that were contributing to expression of OSA, which explains the observed association with OSA itself (e.g., mediated pleiotropy). These individuals may represent a distinct OSA patient subgroup not strongly represented among patients in our large EHR-based sample.

### Additional reasons for lack of validation

A majority of the candidate SNPs we examined did not validate for an association with OSA diagnosis or severity, or associate with other phenotypes in the EHR. As discussed above, this lack of replication may result from limitations in our EHR-based OSA phenotypes or could reflect underlying associations with OSA comorbidities, rather than the disease itself. Ultimately, lack of replication is a common problem in genetic analyses, as initial discovery analyses are more likely to overestimate the true associations (e.g., “Winner’s Curse”) [44]. This can be particularly problematic in candidate gene studies, which tend to rely on smaller sample sizes that are more prone to spurious associations. As described by Varvarigou et al [45], small and underpowered genetic studies, typically lacking replication, are particularly problematic for OSA genetic research. As a result of these issues, it is feasible that a majority of the identified candidate SNPs are false positive associations. Beyond this, the effects of these variants may not generalize to all ancestral populations, as approximately one-third of the SNPs tested in our dataset were identified in populations with different ancestral backgrounds (e.g., Latin American, East Asian). The established heterogeneity in the causes of OSA, including obesity-related pathways [46], specific craniofacial morphologies [47], and physiological mechanisms [48], may also explain the difficulties in replicating associations in large-scale clinical populations like those studies here. Combining individuals with various disease etiologies with potentially distinct genetic influences can obscure underlying associations. Towards this end, careful characterization of OSA cases with respect to disease subtypes and consequences may be required to identify reproducible genetic effects.

### Strengths and limitations

Strengths of this study include the large sample size studied across the two health systems, the detailed information leveraged from electronic health records, and the application of robust association methods to comprehensively evaluate the role of OSA candidate variants.

There are also a number of limitations. A limitation of testing associations between genomic variants and EHR-derived OSA diagnosis is the possibility of undiagnosed OSA among non-cases. Prior studies suggest that a high percentage of individuals referred for sleep studies have undiagnosed OSA [49]; however, these studies are potentially biased given the elevated risk among those referred for testing. In our recent validation study [19], ~ 70% of non-cases had a predicted OSA probability below 20% and fewer than 3% had a predicted probability above 70%, based on the symptomless multi-variable apnea prediction score [50]. Thus, most individuals defined in this study as non-cases are likely true controls. Ultimately, bias introduced by including individuals with undiagnosed OSA in the control group should make it more difficult to identify associations and will not affect the validated associations that we found; however, this could explain negative results. Notably, we complement tests for associations between SNPs and OSA diagnosis with tests between SNPs and OSA severity measures. This should further account for bias related to inaccurate control definitions. Future research using EHR datasets would benefit from studies focused on accurate identification of undiagnosed OSA. Towards this end, obtaining raw sleep study files on large number of patients, both to confirm OSA diagnoses and expand the characterization of quantitative phenotypes, would be beneficial. In addition, while our sample was larger than nearly all of the original studies in which candidate SNPs were identified, the relatively small sample size for specific analyses, particularly among African Americans at VUMC and in quantitative analyses only available in the Geisinger sample, may have limited statistical power for detecting associations. This may be particularly impactful given the expectation that associations in our sample will be smaller than those observed in the original discovery analyses [44]. Finally, to maximize our ability to validate previous associations reported in ancestral populations that are comparable to our study populations, we defined statistical significance for validation of many previously reported associations based on a *p* < 5.0 × 10^− 2^ in analyses of EHR-based OSA diagnosis, which could increase the likelihood of a false positive result. Secondary analyses of quantitative OSA phenotypes and PheWAS analyses were adjusted for multiple comparisons. Ultimately, even at this liberal threshold, a majority of SNPs did not show associations with our endpoints of interest. As such, future work aimed at conducting larger, well-powered genome-wide analyses to assess OSA genetic risk and genetic effects on OSA severity across populations with diverse ancestral backgrounds are warranted.

## Conclusions

This manuscript leverages the breadth of available clinical information in the EHR to comprehensively evaluate the relationships between candidate genetic variants related to OSA and both OSA and non-OSA phenotypes. We validated associations between a small portion of the previously reported OSA candidate SNPs and OSA diagnosis, including variation related to the genes *GABBR1, LEPR* and *MMP-9*, and observed several associations with OSA-related traits from sleep study reports. However, a majority of the candidate SNPs did not show any OSA association in our sample, suggesting these may be false positive results. SNPs related to *APOE* and *HLA* showed stronger associations with non-OSA related phenotypes of hyperlipidemia, cholesterol, thyroid disease and/or celiac disease. Relationships between these conditions and risk for OSA may explain the original associations, or suggest pleiotropic genetic effects. Ultimately, our results reflect the remarkable resources that EHR-derived datasets offer for characterizing the genetic and phenotypic heterogeneity of OSA.

## Supplementary information

**Additional file 1 Figure S1. Inclusion Criteria for Reviewed Studies and Selection of Candidate Variants.** Overview of the systematic literature review procedures, and the process for selecting candidate single nucleotide polymorphisms (SNPs) to test for associations with obstructive sleep apnea (OSA) diagnosis, sleep study report variables and phenome-wide clinical traits. Abbreviations: in/del = insertion/deletion variant, LD = linkage disequilibrium, MAF = minor allele frequency.

**Additional file 2.** Table S1. Clinical Codes Used to Define Sleep Apnea.

**Additional file 3.** Table S2. International Classification of Diseases, Ninth Edition Codes and Corresponding PheCodes Tested for Associations with OSA Candidate Variants.

**Additional file 4.** Table S3. Logical Observation Identifier Names and Codes Mapped Median Laboratory Values Tested for Associations with OSA Candidate Variants.

**Additional file 5.** Table S4. Association Tests for Effects of Candidate SNPs, obtained via Literature Reviews, on Electronic Health Record-derived Obstructive Sleep Apnea (OSA).

**Additional file 6.** Table S5. Results of Interaction Tests for the Effects of Age, Sex and Body Mass Index on Associations between Candidate Variants and Obstructive Sleep Apnoea Diagnosis.

**Additional file 7.** Table S6. Results from Tests for Associations between Obstructive Sleep Apnea Candidate SNPs and Variables Obtained from Sleep Reports at Geisinger.

**Additional file 8.** Table S7. Significant Results from Phenome-wide Studies Testing the Associations between Obstructive Sleep Apnoea Candidate SNPs and Other Clinical Codes and Lab Values.

## Data Availability

The data that support the findings of this study are available from Geisinger and VUMC/BioVU but restrictions apply to the availability of these data, which were used under license for the current study, and so are not publicly available. Data are however available from the authors upon reasonable request and with permission of Geisinger or VUMC/BioVU.

## Abbreviations

OSA: Obstructive Sleep Apnea
EHR: Electronic Health Record
SNP: Single Nucleotide Polymorphism
VUMC: Vanderbilt University Medical Center
PSG: Polysomnography
AHI: Apnea/Hypopnea Index
PheWAS: Phenome-wide association studies
EA: European Americans
AA: African Americans
QC: Quality Control
ICD: International Classification of Diseases
LOINC: Logical Observation Identifier Names and Codes
BMI: Body Mass Index
