# Supplementary figures and images for: Characterization of genetic and phenotypic heterogeneity of obstructive sleep apnea using electronic health records
Source: BMC Med Genomics. 2020 Jul 25;13:105. doi: 10.1186/s12920-020-00755-4 (PMC7382070; doi:10.1186/s12920-020-00755-4)

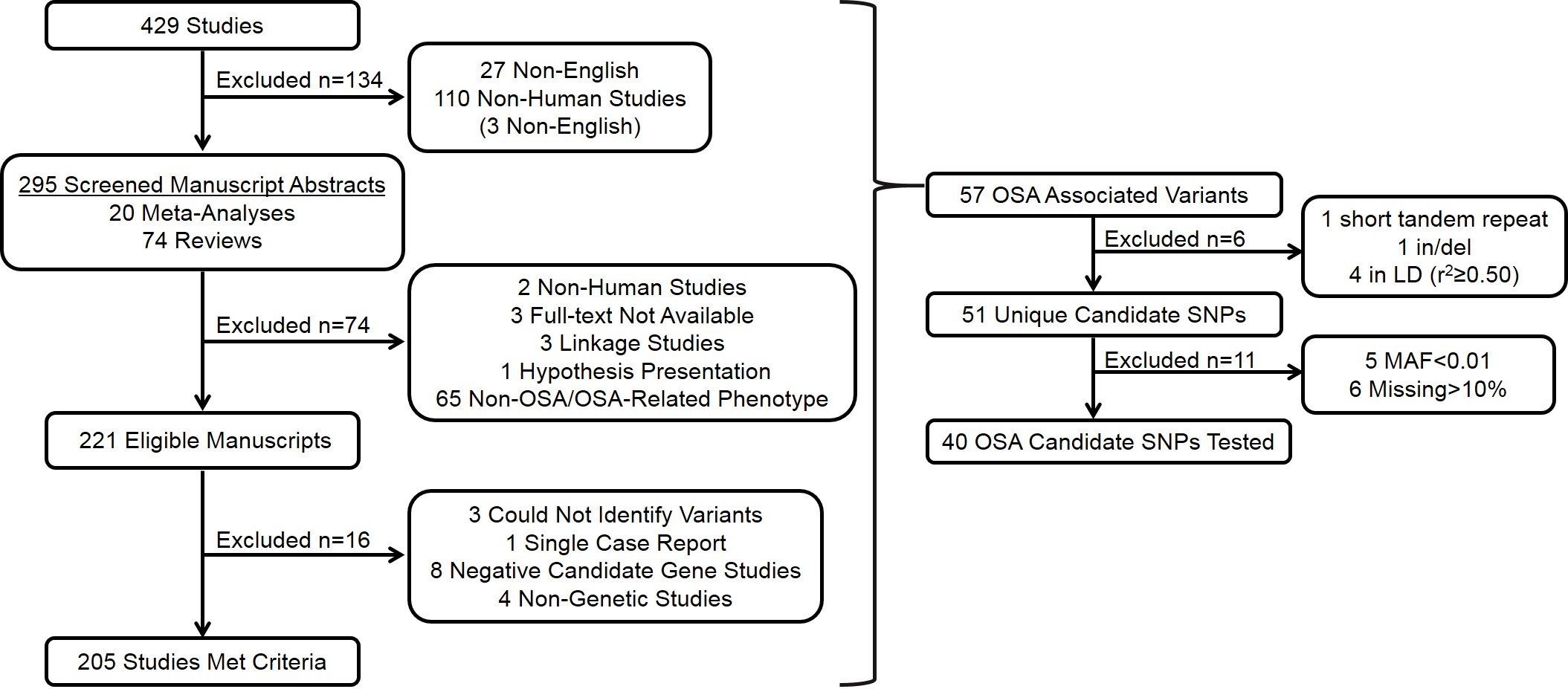

Supplement: Supplementary file 1 — Additional file 1 Figure S1. Inclusion Criteria for Reviewed Studies and Selection of Candidate Variants. Overview of the systematic literature review procedures, and the process for selecting candidate single nucleotide polymorphisms (SNPs) to test for associations with obstructive sleep apnea (OSA) diagnosis, sleep study report variables and phenome-wide clinical traits. Abbreviations: in/del = insertion/deletion variant, LD = linkage disequilibrium, MAF = minor allele frequency. [file 12920_2020_755_MOESM1_ESM.jpg]
